# Associations between different components of fitness and fatness with academic performance in Chilean youths

**DOI:** 10.7717/peerj.2560

**Published:** 2016-10-06

**Authors:** Pedro R. Olivares, Javier García-Rubio

**Affiliations:** 1 Facultad de Educación, Universidad Autonoma de Chile, Talca, Chile; 2 Instituto Superior de Educación Física, Universidad de la República, Rivera, Uruguay; 3 Facultad de Educación, Universidad Autonoma de Chile, Santiago de Chile, Chile

**Keywords:** Physical fitness, Academic achievement, Adolescents, BMI

## Abstract

**Objectives:**

To analyze the associations between different components of fitness and fatness with academic performance, adjusting the analysis by sex, age, socio-economic status, region and school type in a Chilean sample.

**Methods:**

Data of fitness, fatness and academic performance was obtained from the Chilean System for the Assessment of Educational Quality test for eighth grade in 2011 and includes a sample of 18,746 subjects (49% females). Partial correlations adjusted by confounders were done to explore association between fitness and fatness components, and between the academic scores. Three unadjusted and adjusted linear regression models were done in order to analyze the associations of variables.

**Results:**

Fatness has a negative association with academic performance when Body Mass Index (BMI) and Waist to Height Ratio (WHR) are assessed independently. When BMI and WHR are assessed jointly and adjusted by cofounders, WHR is more associated with academic performance than BMI, and only the association of WHR is positive. For fitness components, strength was the variable most associated with the academic performance. Cardiorespiratory capacity was not associated with academic performance if fatness and other fitness components are included in the model.

**Conclusions:**

Fitness and fatness are associated with academic performance. WHR and strength are more related with academic performance than BMI and cardiorespiratory capacity.

## Introduction

Obesity and overweight are one of the major health problems in the world (Malecka-Tendera & Mazur, 2006; Ng et al., 2014) and have been associated with lower scores in academic achievement (Davis & Cooper, 2011), while a high level of physical activity and fitness and low fatness have been related with better cognition (Ruiz et al., 2010b).

In the last few years, the study of academic performance and its relation with fatness, fitness and physical activity has increased drastically (Esteban-Cornejo et al., 2015; Singh et al., 2012). However, evidence regarding the relationship between obesity and academic performance are still inconclusive. Some studies have not proved a direct relationship (Bisset et al., 2012; Rauner et al., 2013), whereas others have found an inverse relationship (Krukowski et al., 2009; Torrijos-Niño et al., 2014). These inconsistencies can be due to the different measures of fatness (usually assessed as Body Mass Index, BMI) and fitness, and their adjustment or lack thereof by different confounders. For example, for fatness analysis, in some studies the BMI was categorized (Roberts, Freed & Mccarthy, 2010; Sardinha et al., 2014), while in others it was treated as continuous variable (Rauner et al., 2013). Some studies have used other fatness variables, such as body fat or visceral fat, measured by whole-body dual energy X-ray absorptiometry (Davis & Cooper, 2011), but without a sufficiently high number of samples due at time of assessment with these measurement techniques. The Waist to Height Ratio (WHR) has been proposed as a better tool than BMI to predict body fat distribution (Hara et al., 2001; Savva et al., 2000) and better discriminator of cardiovascular risk factors (Ashwell, Gunn & Gibson, 2012; Kahn, Imperatore & Cheng, 2005; Savva et al., 2000). However, to the best of our knowledge, the possible association of academic performance and fatness using this index has not been analyzed yet.

An increase in physical activity directly improves cardiorespiratory and musculoskeletal fitness, and this improvement is associated with an increase of academic performance (Ardoy et al., 2014; Coe et al., 2006). On the contrary, lower fitness levels were linked with lower academic performance and a decreased cognitive function associated to perception, memory and cognitive control (Raine et al., 2013). However, more research is needed in order to link different parameters of fitness and academic achievement (Haapala, 2013). Additionally, most studies analyzing fitness and fatness components with academic performance have analyzed this relationship individually for each component (Esteban-Cornejo et al., 2014; Sardinha et al., 2014). Due to the high interdependence between fitness components, fatness indexes, and between fitness and fatness, it is important to analyze all these components in both independent and combined forms in order to know which fitness and fatness components are more relevant in relation to academic performance. Additionally, since socioeconomic status is closely related with academic achievement (Coe et al., 2013) and also with fitness (Freitas et al., 2007), it is important to analyze, in a controlled manner, the association between fitness and academic performance for this factor, among other confounders. Nevertheless, the literature doing this analysis with this adjustment is scarce.

The aim of this study was to analyze the associations between different components of fitness and fatness with academic performance, adjusting the analysis by the main known factors that confound these relationships in a nationally representative Chilean sample. The novelty of this study resides in that it is the first study using the WHR to analyze the relation between fatness and academic performance. Additionally, the association of academic performance, with a complete battery of fitness tests and fatness, has been analyzed in two ways: independently and in combination for each fitness and fatness factor. Additionally, this is the first study performed over a nationally representative sample.

## Methods

### Participants

Data were obtained from the Chilean Standardized System for the Assessment of Educational Quality test (SIMCE) for eighth grade in 2011. SIMCE has national coverage in Chile and is administered by the Ministry of Education to all primary education students attending fourth grade (annually) and eighth grade (biannually), and to all secondary education students attending tenth grade (biannually). The objective of SIMCE is to assess academic achievement in Mathematics, Language, Social Sciences and Natural Sciences by using standardized tests. Some years ago, other academic subjects also began to be evaluated and, since 2010, a representative sample of eighth grade has been evaluated for Physical Education (SIMCE, 2012). The nationally representative SIMCE tests were conducted in all regions of Chile, with the exception of Eastern Island, Juan Fernández archipelago and the Antarctic region. A total of 672 schools and 997 classes participated in the study.

**Figure 1:**
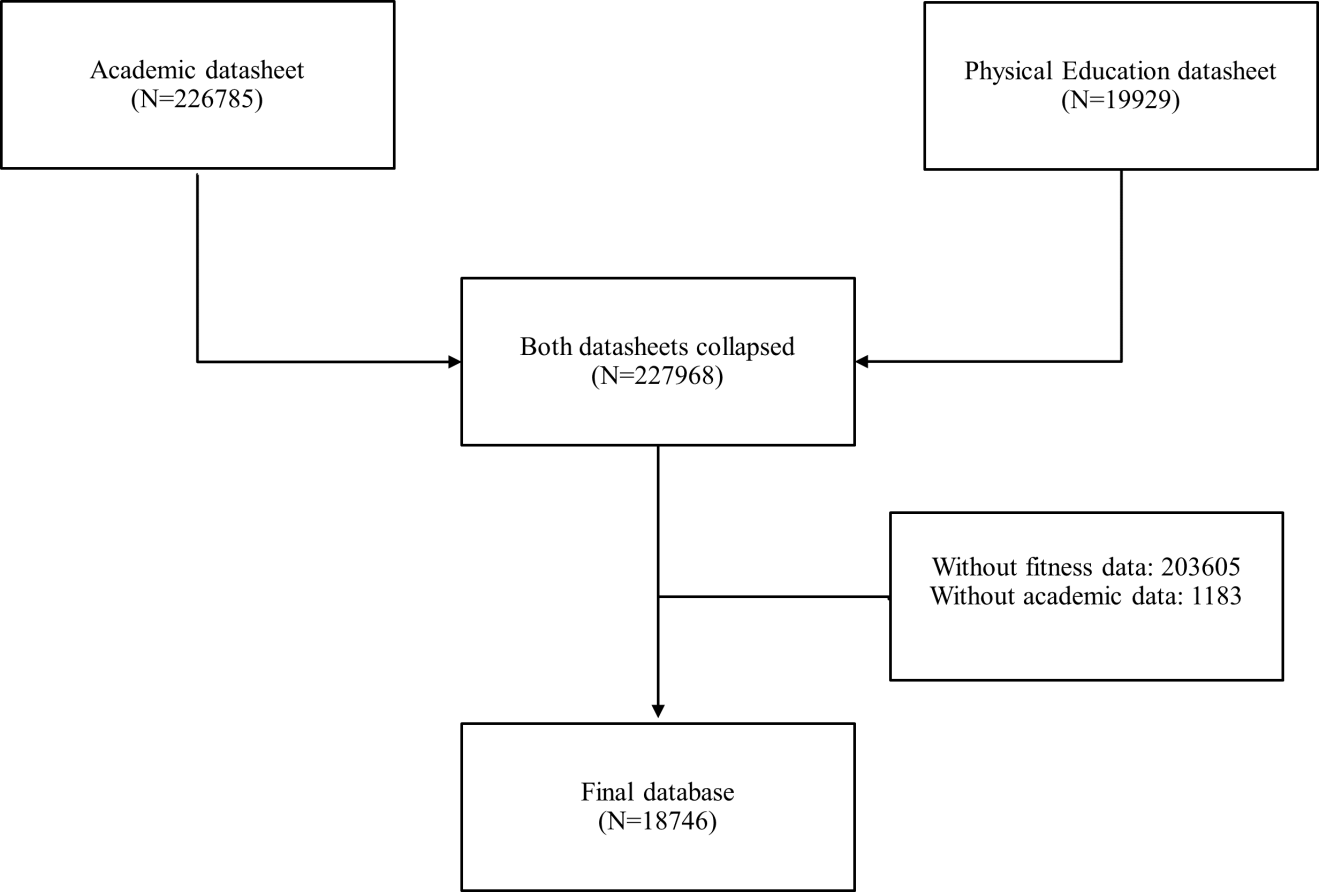
Participants in the study.

In 2011, eighth grade was evaluated for general academic performance (226,785 subjects) and Physical Education (19,929 subjects). Both datasheets were received from SIMCE office and were collated (Fig. 1). A final sample of 18,746 subjects (49% of them were girls) was obtained with data regarding the general academic and physical education evaluations.

### Measures and procedures

SIMCE consists of four standardized tests: Language, Mathematics, Natural Sciences and Social Sciences. Each test, made up of multiple-choice items as well as open-ended questions, is scored on a scale of 0–400. In order to have a single overall academic performance variable, it was computed by using the mean of the *z*-scores from these four tests: Academic *z*-score.

The SIMCE test for Physical Education consists of a fitness assessment using a standardized battery of fitness tests (Ministry of Education, 2013). The full protocol and battery of tests have been previously described (Garber, Sajuria & Lobelo, 2014; Garcia-Rubio et al., 2015), including body composition, cardiorespiratory capacity, strength endurance and flexibility. Anthropometric measures included weight, height, and waist circumference. Fatness indexes, BMI and WHR were calculated through those measures. Cardiorespiratory capacity was measured by using the 20-m shuttle run test and the score was the number of stages completed. Strength endurance was measured by using the push-ups in 30 s for upper limbs, standing broad jump for lower limbs and short crunches for trunk, in which participants have to perform short crunches in a constant rhythm of 50 bpm indicated with a metronome. A single strength endurance variable was computed using the *z*-scores from these tests: Strength *z*-score. For flexibility, the sit and reach test was used. All tests are well validated and highly used for fitness measures (Artero et al., 2011; Castro-Piñero et al., 2010; Ruiz et al., 2010a).

Additionally, SIMCE includes other variables and extra information, such as sex, age, socioeconomic status, school type, and region of the country where the participant lives, which were used as confounder variables in the analyses. School type refers to the three types of schools the participants can attend: (a) Municipal schools, which receive a per-student payment; (b) Subsidized private schools, which receive the same per-student payment and private donations; and (c) Unsubsidized private schools, which operate without public funding (Hsieh & Urquiola, 2006). The socioeconomic status was calculated through cluster analysis and five different groups were formed: (1) Low; (2) Mid-Low; (3) Middle; (4) Mid-High; and (5) High. The variables used in the creation of the groups were the educational level of the mother and father, economic income (self-reported), and vulnerability index (achieved through National Board of Scholar Help and Grants of the Ministry of Education) (SIMCE, 2012).

Prior to testing, every school has to fill a written informed consent and inform parents and students about the procedure (MINEDUC, 2014). The authors of the manuscript comply with the requirements of the Ministry of Education to use the data ethically. The Chilean Law of Sports 19.712, article 5, approved all the aforementioned tests.

Examiners were trained prior to testing by MINEDUC. Fitness tests were carried out in presence of a Physical Education teacher and performed in the school gymnasium or other facilities of the school (Garber, Sajuria & Lobelo, 2014; MINEDUC, 2014). Academic tests were also carried out in school. Assessment took place in November, at the end of the Chilean school year.

### Statistical analysis

First, the amount of data on the database required a preliminary analysis in order to properly organize them and improve the quality of the data. The quantity of schools and classes analyzed implied that some participants missed some of the evaluations. In this study, due to the size of the data, some measurements could be incorrect, or students could give up early in the test or perform the test wrongly. Outliers can occur by chance or due to the measurement error. As a result, outliers bias the means, inflate standard deviation and skew the distribution (Field, 2009). Due to unusual scores in the results of the participants, outliers were cleaned following the procedure state by Hoaglin & Iglewicz (1987).

Descriptive statistics are presented as mean and standard deviations and differences between sexes were tested by *T*-test for independent samples. Partial correlations were adjusted by sex, age, socioeconomic status, region and school type in order to examine the relationships between fitness and fatness variables, and among the variables of academic performance. The associations between the variables of fitness/fatness and academic performance were analyzed by using linear regression models. To explore the combined associations between fitness and fatness with academic performance, three unadjusted and three adjusted models were done. The unadjusted models were: Model 1, using the fitness variables as predictors; Model 2a, using the fitness variables and BMI as predictors; and Model 2b, using the fitness variables and WHR as predictors. In the adjusted models, the variables were controlled by sex, age, socioeconomic status, region and school type. Additionally, linear regression models adjusted for these variables were carried out using each fitness and fatness variable independently. To avoid multicollinearity between BMI and WHR, the combined association of fitness and fatness was analyzed twice, comparing the inclusion of BMI or WHR in separate models (model 2a and 2b). Data analysis was performed using SPSS v.21 software (Inc., Chicago, IL, USA) and statistical significance was set at *p* < 0.05.

## Results

The descriptive characteristics of the participants are shown in Table 1.

**Table 1:** Descriptive characteristics for the sample (values as mean (SD).

	All	Girls	Boys	*p*
Number of Participants (*n*)	18,746	9,118	9,628	
WHR	0.45 (0.06)	0.46 (0.06)	0.45 (0.06)	<0.001
BMI (Kg/m^2^)	22.2 (3.8)	22.7 (3.8)	21.8 (3.7)	<0.001
Push-ups in 30 s (number of repetitions)	12.1 (8.2)	12.0 (7.7)	12.2 (8.7)	0.136
Standing broad jump (cm)	151.1 (31.7)	131.6 (22.3)	169.6 (28)	<0.001
Short crunches (number of repetitions)	21.4 (6.2)	20.1 (7.1)	22.7 (4.9)	<0.001
Sit and reach (cm)	30.6 (8.4)	33.2 (7.8)	28.1 (8.1)	<0.001
20-m shuttle run (ml kg^−1^ min^−1^)	42.4 (7.8)	38.2 (6.1)	46.2 (7.2)	<0.001
Academic performance Scores				
Language (0–400)	254.5 (49.4)	259.7 (47.5)	249.6 (50.6)	<0.001
Mathematics (0–400)	258.4 (48.3)	254.9 (48)	261.7 (48.4)	<0.001
Social sciences (0–400)	259.2 (48.4)	254.6 (46.8)	263.5 (49.5)	<0.001
Science (0–400)	262.3 (50.8)	260.2 (48.9)	264.2 (52.6)	<0.001
Age (years)	13.8 (0.7)	13.8 (0.7)	13.9 (0.8)	<0.001

**Notes.**

WHR Waist to Height Ratio BMI Body Mass Index

*P* of *T*-test.

Fatness indexes were higher for girls, while fitness performance was higher for boys, except in the sit and reach test (all *p* < .001 except push-ups). In academic performance, girls scored higher in Language test while boys scored higher for Mathematics, Social sciences and Science (all *p* < .001).

Table 2 presents the partial correlations of the fitness and fatness components after adjustment for sex, age, socioeconomic status, region and type of school. WHR was negatively correlated with all fitness components, while BMI was negatively correlated with cardiorespiratory capacity and strength (all *p* < 0.001).

**Table 2:** Partial correlation coefficients between physical fitness and fatness components.

	BMI	Cardiorespiratory capacity	Strength *z*-score	Flexibility
WHR	0.835^*^	−0.318^*^	−0.309^*^	−0.050^*^
BMI		−0.321^*^	−0.281^*^	0.012
Cardiorespiratory capacity			0.382^*^	0.145^*^
Strength *z*-score				0.236^*^

**Notes.**

Analyses were adjusted for age, sex, socioeconomic status, region and type of school.

WHR Waist to Height Ratio BMI Body Mass Index

* *p* < .001.

Table 3 shows that all academic performance variables were positively associated with each other after adjustment for age, sex, socioeconomic status, region and type of school.

**Table 3:** Partial correlation coefficients between academic performance variables.

	Language	Social sciences	Science	Academic *z*-score
Mathematics	0.623^*^	0.593^*^	0.665^*^	0.839^*^
Language		0.632^*^	0.686^*^	0.862^*^
Social sciences			0.652^*^	0.841^*^
Science				0.878^*^

**Notes.**

Analyses were adjusted for age, sex, socioeconomic status, region and type of school.

* *p* < .001.

Table 4 shows the independent associations of each fitness and fatness component with academic performance, after adjustment for age, sex, socioeconomic status, region and type of school. All fitness and fatness components were statistically associated with all academic performance variables (all *p* < .001), except the cardiorespiratory capacity and body mass index with social sciences. Strength component showed the highest standardized *β* for most academic variables. Standardized *β* was higher for WHR than BMI for all academic variables.

**Table 4:** Independent associations between physical fitness and fatness with academic performance.

	Mathematics				Languaje				Social sciences				Science				Academic *z*-score			
			CI 95%				CI 95%				CI 95%				CI 95%				CI 95%	
Predictor variables	*β*	B	Lower	Upper	*β*	B	Lower	Upper	*β*	B	Lower	Upper	*β*	B	Lower	Upper	*β*	B	Lower	Upper
Cardiorespiratory capacity	**0.06**	0.38	0.29	0.47	**0.03**	0.22	0.12	0.32	0.01	0.00	−0.08	0.10	**0.02**	0.15	0.05	0.24	**0.03**	0.00	0.01	0.01
Strength *z*-score	**0.12**	8.00	7.22	9.18	**0.08**	5.75	4.71	6.80	**0.04**	3.15	2.14	4.16	**0.09**	6.70	5.65	7.76	**0.09**	0.12	0.10	0.13
Flexibility	**0.08**	0.46	0.38	0.54	**0.07**	0.45	0.37	0.53	**0.04**	.025	.018	.033	**0.07**	0.42	0.34	0.51	**0.07**	0.00	0.00	0.01
BMI	**−0.03**	−0.35	−0.51	−0.18	**−0.04**	−0.59	−0.76	−0.41	−0.00	−0.08	−0.25	0.08	**−0.03**	−0.45	−0.63	−0.28	**−0.03**	−0.00	−0.10	−0.00
WHR	**−0.05**	−43.93	−55.23	−32.62	**−0.06**	−57.18	−69.24	−45.11	**−0.03**	−29.02	−40.63	−17.41	**−0.05**	−52.30	−64.45	−40.14	**−0.05**	−0.91	−1.12	−0.70

**Notes.**

Analyses were adjusted for age, sex, socioeconomic status, region and type of school.

*β* Standardized beta coefficient B Beta coefficient SE Standard error for beta coefficient CI Confidence Interval WHR Waist to Height Ratio BMI Body Mass Index **Bold** *p* < 0.01

**Table 5:** Combined associations between physical fitness and fatness with academic performance.

	Mathematics				Languaje				Social sciences				Science				Academic *z*-score			
			CI 95%				CI 95%				CI 95%				CI 95%				CI 95%	
Predictor variables	*β*	B	Lower	Upper	*β*	B	Lower	Upper	*β*	B	Lower	Upper	*β*	B(SE)	Lower	Upper	*β*	B	Lower	Upper
Unadjusted model 1																				
Cardiorespiratory capacity	**0.05**	0.30	0.20	0.40	−0.02	−0.13	−0.24	−0.03	**0.02**	0.17	0.07	0.28	0.00	0.06	−0.04	0.17	0.01	0.00	0.01	0.01
Strength *z*-score	**0.14**	10.18	9.04	11.32	**0.06**	4371	3.54	5.88	**0.10**	7.23	6.07	8.38	**0.12**	9.22	8.01	10.42	**0.13**	0.16	0.14	0.18
Flexibility	**0.05**	0.32	0.23	0.40	**0.10**	0.61	0.53	0.70	**0.04**	0.11	0.03	0.20	**0.05**	0.33	0.25	0.42	**0.06**	0.00	0.01	0.01
Unadjusted model 2a																				
Cardiorespiratory capacity	**0.04**	0.25	0.14	0.35	**−0.04**	−0.25	−0.36	−0.14	**0.02**	0.14	0.03	0.25	−0.00	−0.02	−0.13	0.08	0.00	0.00	−0.01	0.01
Strength *z*-score	**0.14**	9.82	8.66	10.97	**0.05**	3.77	2.59	4.96	**0.10**	6.93	5.76	8.10	**0.11**	8.56	7.34	9.78	**0.12**	0.15	0.13	0.17
Flexibility	**0.05**	0.33	0.24	0.41	**0.10**	0.64	0.55	0.72	**0.02**	0.12	0.04	0.21	**0.05**	0.35	0.26	0.44	**0.06**	0.00	0.01	0.01
BMI	**−0.03**	−0.38	−0.58	−0.18	**−0.07**	−0.98	−1.18	−0.78	**−0.02**	−0.29	−0.49	−0.09	**−0.05**	−0.68	−0.88	−0.47	**−0.04**	−0.01	−0.01	−0.01
Unadjusted model 2b																				
Cardiorespiratory capacity	**0.03**	0.20	0.09	0.30	**−0.04**	−0.27	−0.38	−0.16	0.01	0.08	−0.01	0.19	−0.01	−0.06	−0.17	0.04	−0.00	0.00	−0.01	0.01
Strength *z*-score	**0.13**	9.16	8.00	10.31	**0.04**	3.23	2.04	4.41	**0.09**	6.22	5.05	7.38	**0.10**	7.90	6.68	9.12	**0.11**	0.13	0.11	0.16
Flexibility	**0.05**	0.30	0.22	.039	**0.10**	0.59	0.51	0.68	0.01	0.10	0.02	0.18	**0.05**	0.32	0.23	0.40	**0.06**	0.00	0.01	0.01
WHR	**−0.07**	−67.35	−80.63	−54.07	**−0.11**	−98.54	−112.17	−84.90	**−0.07**	−65.23	−78.67	−51.78	**−0.09**	−87.15	−101.18	−73.13	**−0.09**	−1.59	−1.83	−1.34

**Notes.**

*β* Standardized beta coefficient B beta coefficient SE Standard error for beta coefficient CI Confidence Interval WHR Waist to Height Ratio BMI Body Mass Index **Bold** *p* < 0.001

**Table 6:** Combined associations between physical fitness and fatness with academic performance.

	Mathematics				Languaje				Social sciences				Science				Academic *z*-score			
			CI 95%				CI 95%				CI 95%				CI 95%				CI 95%	
Predictor variables	*β*	B	Lower	Upper	*β*	B	Lower	Upper	*β*	B	Lower	Upper	*β*	B	Lower	Upper	*β*	B	Lower	Upper
Adjusted model 1																				
Cardiorespiratory capacity	0.01	0.08	−0.02	0.17	0.00	−0.00	−0.11	0.10	**−0.02**	−0.13	−0.24	−0.03	−0.02^*^	−0.13	−0.23	−0.02	−0.00	−0.00	−0.01	0.01
Strength *z*-score	**0.10**	6.91	5.83	7.99	**0.06**	4.65	3.50	5.81	**0.04**	3.05	1.93	4.16	**0.08**	6.23	5.07	7.39	**0.08**	0.10	0.08	0.12
Flexibility	**0.05**	0.32	0.24	0.40	**0.06**	0.37	0.28	0.45	**0.03**	0.22	0.14	0.30	**0.05**	0.33	0.24	0.41	**0.06**	0.00	0.01	0.01
Adjusted model 2a																				
Cardiorespiratory capacity	0.01	0.07	−0.02	0.18	−0.01	−0.07	−0.17	0.04	**−0.02**	−014	−0.25	−0.03	**−0.03**	−0.17	−0.28	−0.06	−0.01	−0.00	−0.00	0.00
Strength *z*-score	**0.10**	6.94	5.84	8.04	**0.06**	4.09	2.92	5.27	**0.04**	2.99	1.85	4.12	**0.08**	5.86	4.68	7.05	**0.08**	0.10	0.08	0.12
Flexibility	**0.05**	0.32	0.24	0.40	**0.06**	0.39	0.30	0.47	**0.04**	0.22	0.14	0.30	**0.06**	0.34	0.26	0.43	**0.06**	0.00	0.01	0.01
BMI	0.01	0.01	−0.16	0.18	**−0.03**	−0.45	−0.64	−0.27	−0.03	−0.03	−0.21	0.14	**−0.02**	−0.29	−0.48	−0.10	**−0.01**	−0.00	−0.01	−0.01
Adjusted model 2b																				
Cardiorespiratory capacity	0.01	0.04	−0.05	0.14	−0.01	−0.09	−0.19	0.02	**−0.03**	−0.18	−0.29	−0.08	**−0.03**	−0.20	−0.31	−0.09	**−0.02**	−0.01	−0.49	0.00
Strength *z*-score	**0.10**	6.59	5.48	7.69	**0.05**	3.79	2.60	4.97	**0.04**	2.51	1.37	3.65	**0.07**	5.48	4.29	6.67	**0.07**	0.09	0.07	0.11
Flexibility	**0.06**	0.33	0.25	0.40	**0.06**	0.38	0.29	0.46	**0.04**	0.22	0.14	0.31	**0.05**	0.34	0.25	0.43	**0.06**	−0.01	−0.01	−0.01
WHR	**−0.02**	−16.17	−28.33	−4.02	**−0.05**	−44.42	−57.43	−31.42	**−0.03**	−25.90	−38.45	−13.35	**−0.04**	−37.98	−51.08	−24.88	**−0.04**	−0.62	−0.84	−0.39

**Notes.**

Analyses were adjusted for age, sex, socioeconomic status, region and type of school.

*β* Standardized beta coefficient B beta coefficient SE Standard error for beta coefficient CI Confidence Interval WHR Waist to Height Ratio BMI Body Mass Index **Bold** *p* < 0.01

* *p* < 0.05.

Tables 5 and 6 presents the combined associations of each fitness and fatness component with academic performance by using three unadjusted and three adjusted independent models (confounder variables for adjusted models were sex, age, socioeconomic status, region and type of school). Model 1 included only combined fitness components, Model 2a included the combined fitness components and BMI as fatness component, while Model 2b included the combined fitness components and WHR as fatness component. Strength *z*-score was the component more related with academic performance in almost all combined association models, while cardiorespiratory was the least related. Between the two analyzed fatness components, WHR was always statistically associated with all academic variables and with higher standardized *β* than BMI. When confounding variables were included in the analysis, BMI was not statistically associated with Mathematics and Social sciences, while it was associated with all academic variables in the unadjusted model.

## Discussion

Results in this study suggest that both fitness and fatness indexes have an association with academic performance. Nevertheless, available literature suggests that there are negative associations between fitness and fatness in some studies (Davis & Cooper, 2011; Kristjánsson, Sigfúsdóttir & Allegrante, 2010; Roberts, Freed & Mccarthy, 2010), and no association in others (Bisset et al., 2012; London & Castrechini, 2011; Van Dusen et al., 2011). These studies measured fatness or weight status through BMI, having different results due to the differences in the quantification of BMI. Some studies used BMI as categorical variable and other studies as continuous variable to prevent loss of information (Sardinha et al., 2014). Additionally, Van Dusen et al. (2011) found that, in boys, low BMI was associated with lower academic performance relative to moderate BMI, but not to high BMI, suggesting that there is no linear association between these variables after covariate adjustment. To analyze it, we have explored non-linear association between these variables but, although quadratic analysis fit the model, the R^2^ was practically equal than the linear model.

In the present study, we added the WHR as a measure of fatness. It is more associated with central adiposity and metabolic syndrome than BMI (Lee et al., 2008; Savva et al., 2000), but to the best of our knowledge, there are no previous studies analyzing its relationship with academic performance. Results highlighted the higher association of WHR than BMI to predict academic performance, and its results were more robust in the analyses performed. Moreover, WHR had a significant impact on academic performance despite the confounders controlled analysis. Notwithstanding the aforementioned, BMI changed its association according to the confounders. This suggests that WHR appears to be more independent of the context to assess the relationship between fatness and academic performance due to its significance across different model analysis. The mechanisms that explain the association of fatness and academic performance have been stated in two pathways: physiological, disturbing memory functioning, and psychosocial, where self-esteem or discrimination results in behavior problems and affect the school performance of children (Sardinha et al., 2014).

For fitness components, strength was the variable most closely associated with the academic performance in all models with the exception of Language in the unadjusted combined analysis, and it had a higher standardized *β* than fatness component. As it happened with BMI, results of previous studies relating strength with fitness performance are controversial. Some studies found association between muscular strength and academic performance (Coe et al., 2013; Eveland-Sayers et al., 2009; Van Dusen et al., 2011), while some studies did not found any association between changes in muscular strength and academic performance (Castelli et al., 2007; Chen et al., 2013; Edwards, Mauch & Winkelman, 2011). In this study, strength was assessed as the mean of strength tests, as a single measure of overall strength, while this fitness component is usually related with academic performance using only single tests.

Previous studies have stated cardiorespiratory capacity as the fitness component most related with academic performance (Van Dusen et al., 2011) and other studies have associated cardiorespiratory capacity and academic performance positively (Esteban-Cornejo et al., 2015; Rauner et al., 2013; Sardinha et al., 2014; Torrijos-Niño et al., 2014). Our results indicate that it was independently associated with most academic variables, but when it was combined with fatness and the other fitness components, this association was not maintained.

For flexibility, results indicate that it has both, independent and combined significant association with all analyzed academic scores. However, most of previous studies are focused on cardiorespiratory and strength components, and few studies have included flexibility as fitness component in the analysis of this relationship. Other studies have found similar results (Van Dusen et al., 2011) but the mechanism that explain the relation between flexibility and academic achievement is unclear. We hypothesize this could be due to physical activity related with higher flexibility (Marques et al., 2015) and better academic achievement (Maher et al., in press), so indirectly people with higher flexibility could have better academic achievement, but more research is needed.

In terms of relevance of fitness and fatness in academic performance, associations in this study are low, but higher that findings in previous studies (*R*^2^ < 0.1). These low associations are a probable cause of mixed findings in the literature presumably due to the much lower sample size than the present work. Even so, schools have to stimulate activities that promote other benefits of physical education and not only mere weight control in order to increase academic performance (Van Dusen et al., 2011).

In the analysis, we included as moderators some variables that affect the relationship between fitness and fatness with academic performance, as literature suggests (Roberts, Freed & Mccarthy, 2010; Sardinha et al., 2014), such as socioeconomic status, school type or region. In fact, socioeconomic status is a moderator of poor fitness (London & Castrechini, 2011) and previous studies have shown it is a factor more associated with academic achievement than fitness (Coe et al., 2013). In our study, socioeconomic status had the highest standardized *β* for all adjusted models. School type in Chile is strongly related with the economic status, due to the fees that families have to pay to join the establishment. Sex and age of the participants have been included as confounders, too. Girls usually have higher prevalence of obesity than boys (Garcia-Rubio et al., 2015; Mond et al., 2007). All scholars were 8th graders at the moment of the assessment, but the age might be different. There could be children with a lower academic performance but a higher fitness performance, due to the Relative Age Effects (Roberts et al., 2012; Wattie et al., 2014) because they had failed their course, so the age at the moment of evaluation was also considered as confounder.

Results need to be interpreted with caution. We have carried out a cross-sectional study, which can only indicate association, not causality. We have tried to eliminate other limitations addressed by available literature, such as the use of standardized academic outcomes, the use of an alternative index to assess fatness and other socioeconomic indicators (Roberts, Freed & Mccarthy, 2010; Sardinha et al., 2014). In addition, the estimation of VO2 was calculated through numbers of stages completed which could attenuate the association for cardiorespiratory fitness. The use of shuttles would provide a better picture of aerobic fitness level but the SIMCE dataset did not include this data. The study also has several strengths, such as the representative of the sample due to its size, or the uses of standardized tests to assess fitness and fatness. The next step in the study of academic performance and fitness relationships must be the use of longitudinal designs in order to establish causality.

## Conclusion

Fitness and fatness are associated with academic performance. WHR and strength are more related with academic performance than BMI and cardiorespiratory capacity, after adjustment for age, sex, socioeconomic status, region and type of school.

## Additional Information and Declarations

### Data Availability

The following information was supplied regarding data availability: The raw data is the property of the Government of Chile, which is public at the “Bases de datos nacionales, Ministerio de Educación, Gobierno de Chile”: http://www.agenciaeducacion.cl/simce/bases-de-datos-nacionales/. For the specific dataset used in this study, please contact the office which manages all data: contacto@agenciaeducacion.cl.
